# Optimized fixation of actin filaments for improved indirect immunofluorescence staining of rickettsiae

**DOI:** 10.1186/s13104-019-4699-9

**Published:** 2019-10-16

**Authors:** Monika Danchenko, Lucia Csaderova, Pierre Edouard Fournier, Zuzana Sekeyova

**Affiliations:** 1 Biomedical Research Center, Slovak Academy of Sciences, Dúbravská cesta 9, 845 05 Bratislava, Slovakia; 2 UMR VITROME, AMU, IRD SSA, AP-HM, IHU Mediterranee Infection, Marseille, France

**Keywords:** Immunofluorescence assay, Rickettsiae, Cytoskeleton fixation, Cell culture, Actin-based motility

## Abstract

**Objective:**

The objective was to investigate fixative solutions: 3.7% formaldehyde, 4% paraformaldehyde, 4% paraformaldehyde in the cytoskeletal buffer and 4% paraformaldehyde in PHEM buffer (containing PIPES, HEPES, EGTA and MgCl_2_), applicable for immunofluorescence assay.

**Results:**

Herein we optimized this serological technique, testing four fixative solutions, for the sensitive detection of rickettsial antigens, and preservation of intracellular structures of the host cells, particularly filamentous actin. Rickettsial antigens were presented equally well both with formaldehyde and all paraformaldehyde-based fixations, but only protocol with 4% paraformaldehyde in PHEM buffer allowed accurate imaging of actin filaments, and simultaneously allows monitoring of rickettsiae using actin-based motility during infection inside the host cells.

## Introduction

Members of the genus *Rickettsia* are Gram-negative strictly intracellular Alphaproteobacteria, multiplying by binary fission in the cytoplasm and nuclei of infected host cells [13, 19]. Rickettsiae are arthropod-vectored pathogens, causing from mild to very severe diseases in animals and humans, which are widely distributed throughout the world [2, 20]. Serology using indirect immunofluorescence assay (IFA) is considered as the “gold standard” for diagnostic confirmation [16]. Molecular detection using polymerase chain reaction (PCR) demonstrates the presence of the bacterium itself. However, a positive PCR does not predict the viability of bacteria and does not provide an accurate indication of their localization in tissues.

According to phylogenomic studies, *Rickettsia* spp. are divided into spotted fever group (SFG), typhus group (TG), transitional group (TRG) and ancestral group (AG) [8, 18]. SFG rickettsiae and one TG species, *R. typhi*, can polymerize actin of eukaryotic host cells and use actin-based motility for intra- and intercellular spread. Assembly of the filamentous actin “tail” transports the pathogen through the host cytosol and to cell membranes [9, 11]. Therefore, actin “tails” appear as adequate target to be visualized during active rickettsial infection in the eukaryotic cell.

We would like to investigate four fixative solutions: 3.7% formaldehyde (FA), 4% paraformaldehyde (PFA), 4% paraformaldehyde in cytoskeletal buffer (PFA + CB) [24] and 4% paraformaldehyde in PHEM buffer (PFA-PHEM) [25], with the aim to optimize preservation of rickettsial antigens, the morphology of the host cell constituents, and to visualize dispersal of actin “tails”.

## Main text

### Methods

#### Reagents

Paraformaldehyde, MES hydrate, EGTA, HEPES, Triton X-100 and Tween 20 were from Sigma-Aldrich (USA). Blotting-grade blocker non-fat dry milk was from Bio-Rad (USA). Potassium chloride, magnesium dichloride, potassium dihydrogen phosphate, potassium hydrogen phosphate, sodium hydrogen phosphate, sucrose and formaldehyde (FA) were from Slavus (Slovakia). Sodium chloride was from CentralChem (Slovakia). Potassium hydroxide and sodium hydroxide were from Lachema (Czech Republic). PIPES was from AppliChem (Germany). Glutamic acid was from Duchefa Biochemie (Netherlands). Dulbecco’s Modified Eagle’s Medium, trypsin 10x with versene and fetal bovine serum were from Lonza (Switzerland). Vectashield Mounting Medium with DAPI was from Vector Laboratories (UK). We handled all chemical compounds according to the recommendations in their safety data sheets.

#### Antibodies

Rhodamine Red-X goat anti-rabbit IgG (H + L) polyclonal secondary antibody was from Invitrogen (reference number R6394, lot: 1402199, made in the USA). Alexa Fluor 488 phalloidin was from Invitrogen (reference number A12379, lot: 1378369, made in the USA). We prepared primary anti-*Rickettsia akari* and anti-*Rickettsia slovaca* rabbit antibodies (in serum) in our experimental animal facility at Biomedical Research Center.

#### Bacterial species and host cell line

The bacterial species used in the study were *Rickettsia akari*, strain MK (Kaplan, ATCC VR-148) and *Rickettsia slovaca*, strain 13-B (ATCC VR-1639). Bacteria were propagated in confluent monolayers of Vero cell line (African green monkey kidney cells, ATCC CCL-81), maintained in RPMI 1640 medium supplemented with 2 mM l-glutamine, 25 mM HEPES and heat-inactivated fetal bovine serum at 34 °C in a humidified 5% CO_2_ incubator. Rickettsiae were purified by isopycnic density gradient centrifugation [1, 29]. Infections of the host cells were initiated from frozen rickettsial stocks stored in SPG buffer (0.218 M sucrose, 3.76 mM KH_2_PO_4_, 7.1 mM K_2_HPO_4_ and 4.9 mM potassium glutamate). The concentration of the rickettsiae was determined by Real-Time quantitative PCR as described earlier [5].

#### Immunofluorescence assay

One day prior to the experiment, host cells were trypsinized and seeded onto 12-well plates appropriate for tissue culture, containing round glass coverslips and supplemented RPMI medium. Cultured to semi-confluence, Vero cells were infected with rickettsiae (multiplicity of infection, MOI = 25). After 48 h, the medium was aspirated, and infected cells were gently washed twice with pre-warmed PBS (0.13 M NaCl, 0.01 M Na_2_HPO_4_, 1.5 mM KH_2_PO_4_, pH 7.2).

Next, four different fixatives were added for comparison: 3.7% formaldehyde in PBS pH 7.2 (FA), 4% paraformaldehyde in PBS pH 7.2 (4% PFA), 4% paraformaldehyde in cytoskeleton buffer (4% PFA + CB) containing 10 mM MES hydrate pH 6.1, 1 mM KCl, 3 mM MgCl_2_, 2 mM EGTA and 0.32 M sucrose [24] and 4% paraformaldehyde in PHEM buffer (PFA-PHEM) containing 60 mM PIPES, 25 mM HEPES, 10 mM EGTA, 2 mM MgCl_2_, pH 6.9 [25]. We allowed fixation at 37 °C for 15 min in the case of 3.7% FA and 4% PFA, or at room temperature for 10 min in the case of 4% PFA-PHEM and 4% PFA + CB.

After removing the fixatives, cells on the coverslips were washed three times with PBS, moderately agitating for 2 min on a shaker. For permeabilization, we added 0.1% Triton X-100 in PBS to each well and cells were incubated at room temperature for 7 min. This step was followed by washing with PBS as described above. Blocking was performed at 37 °C for 1 h with 5% milk solution in PBS.

After aspiration of the blocking solution, cells on the coverslips were washed, and intracellular bacteria were subsequently probed with a primary antibody. Rabbit serums containing polyclonal anti-*Rickettsia* antibodies were diluted 1:100 with 5% milk in PBS and samples were incubated at 37 °C for 1 h. Each coverslip was then washed three times to remove unbound antibodies by adding 0.1% Tween 20 in PBS, moderately agitating for 5 min on a shaker. For visualization of rickettsiae, we applied fluorophore-conjugated goat anti-rabbit secondary antibody diluted 1:1000 into 5% milk in PBS. Subsequently, cell cultures were incubated at 37 °C for 1 h in the dark. Afterward, the coverslips were thoroughly washed with 0.1% Tween 20 in PBS.

To localize filamentous actin, the cells were probed with fluorescently tagged phalloidin, diluted into 5% milk in PBS according to the manufacturer’s instruction. Plates were incubated at room temperature for 30 min in the dark. After three further prolonged washing with 0.1% Tween 20 in PBS, coverslips were air-dried and counterstain with mounting medium containing DAPI. Specimens were stored at ‒ 20 °C.

#### Controls

Slides with infected cells treated only with 5% milk in PBS and conjugated secondary antibody mixture were included to ensure the absence of nonspecific reactivity with the sample. Uninfected cells treated with both primary and secondary antibodies were used as negative controls to show the specificity of the primary antibody used for detection of rickettsiae.

#### Fluorescent microscopy

Images of stained samples were acquired by a confocal fluorescence microscope (Zeiss LSM 510 META, Germany) in multi-track scanning mode using Plan Apochromat 100×/1.40 oil immersion objective. The microscope settings were as follows: excitation of 420 nm, band pass filter 420–480 nm to visualize nuclei, excitation 488 nm, band pass filter 505–550 nm for actin (fluorophore Alexa Fluor 488), excitation 543 nm and long pass filter over 560 nm for rickettsia (fluorophore Rhodamine Red-X). Brightness and contrast were adjusted, if necessary, by LSM Image Browser software from Zeiss.

### Results

**Fig. 1:**
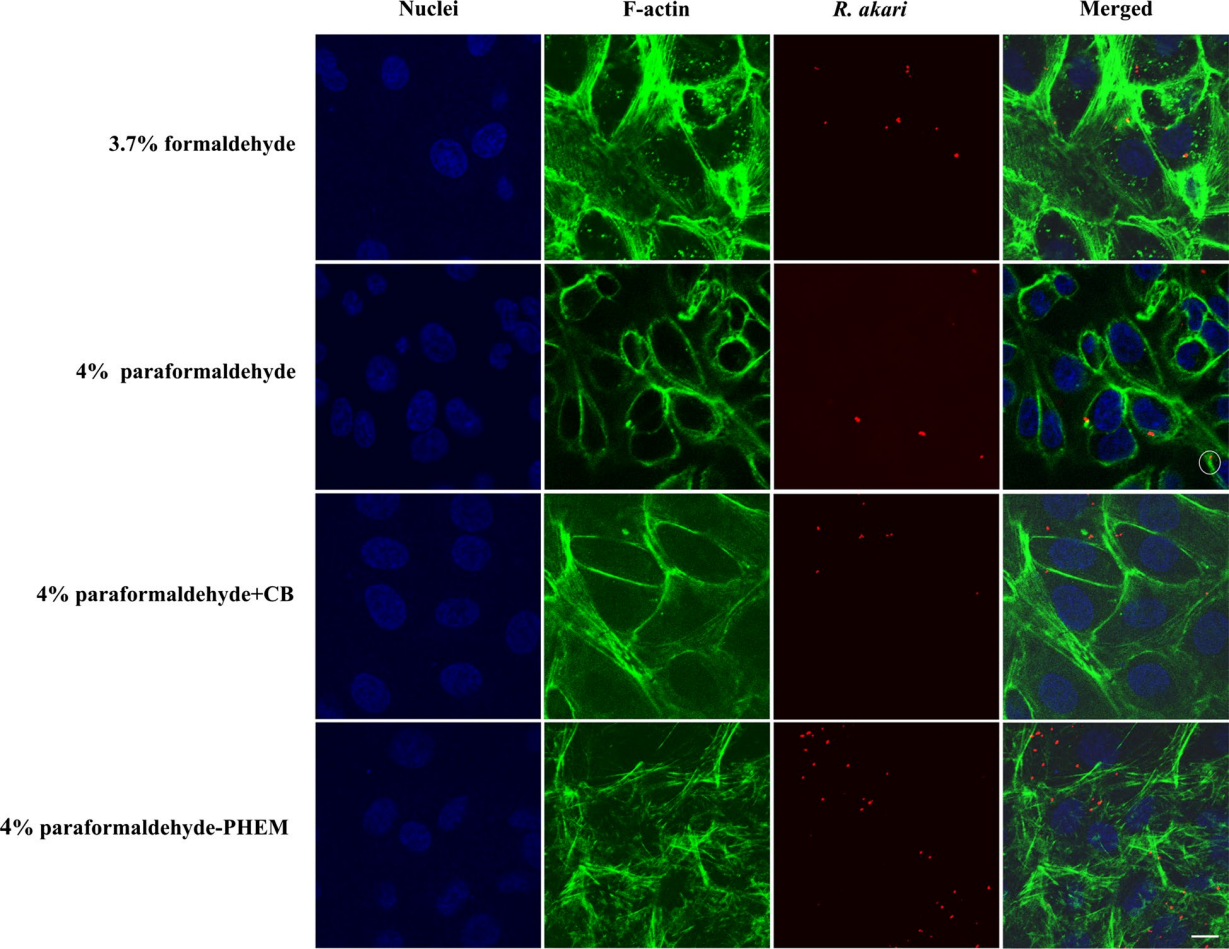
Indirect immunofluorescent staining of Vero cells infected with *R. akari* and comparison of four fixative solutions. We applied an anti-*R. akari* rabbit serum as primary antibody and goat anti-rabbit Rhodamine Red-X secondary antibody (red fluorescence signal) to visualize bacteria. To show the overall shape of the host cells and actin polymerization by *R. akari* (in a white circle), we employed Alexa Fluor 488 phalloidin for F-actin staining (green fluorescence signal). We compared 3.7% formaldehyde, 4% paraformaldehyde, 4% paraformaldehyde in the cytoskeletal buffer and 4% paraformaldehyde in PHEM buffer as fixatives. For nuclear counterstaining, mounting medium with DAPI was used (blue fluorescence signal). Scale bar represents 10 µm

**Fig. 2:**
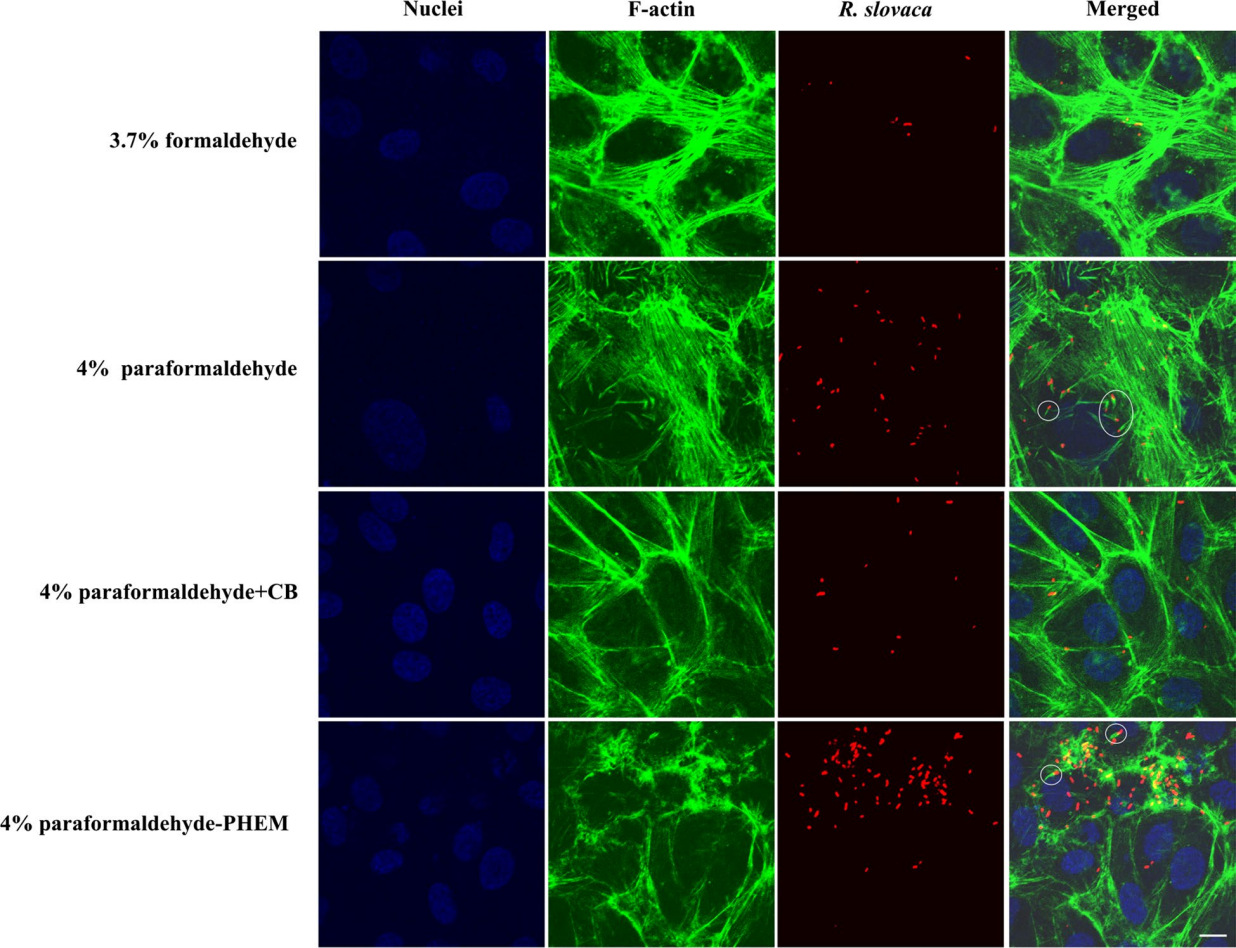
Indirect immunofluorescent staining of Vero cells infected with *R. slovaca* and comparison of four fixative solutions. We employed an anti-*R. slovaca* rabbit serum as primary antibody and goat anti-rabbit Rhodamine Red-X secondary antibody (red fluorescence signal) to probe bacteria. To visualize “comet-tail” formation (in white circles), we applied Alexa Fluor 488 phalloidin for F-actin staining (green fluorescence signal). *Rickettsia slovaca* formed actin-tails at one pole of the bacterium around 5 µm in length. Likewise, to infected Vero cells with a related intracellular bacterium *R. akari*, we paralleled fixation step with 3.7% formaldehyde, 4% paraformaldehyde, 4% paraformaldehyde in the cytoskeletal buffer and 4% paraformaldehyde in PHEM buffer. We counterstained nuclei with mounting medium containing DAPI (blue fluorescence signal). Scale bar represents 10 µm

We used a range of available fluorophores which facilitate the simultaneous localization of as many as three structures/epitopes of interest in a single cell: *Rickettsia* sp. (Rhodamine Red), filamentous actin (Alexa Fluor 488) and host cell nuclei (DAPI). Representative results from the comparison of the suitability of aldehyde fixatives for infected Vero cells in vitro with two different species of the genus *Rickettsia*, namely *R. akari* and *R. slovaca*, are shown in Figs. 1 and 2, respectively.

#### A rickettsial antigen is preserved equally well with formaldehyde and paraformaldehyde-based fixation

We detected selective and robust staining for rickettsial antigens with both tested species. We did not observe relevant differences in the quality of the micrographs, which would be attributed to the use of either formaldehyde or paraformaldehyde. Co-localized signal (yellow reaction product—equals a red signal of rickettsiae and green signal of filamentous actin) was demonstrated in almost all captured merge images, providing proof of intracellular localization of bacteria.

Additionally, we also prepared appropriate controls. Uninfected Vero cells probed with both primary and conjugated secondary antibodies gave no rhodamine red response. Likewise, slides with infected cells but without primary antibodies against *R. akari* or *R. slovaca* displayed no specific red signal.

#### Fixation with PFA-PHEM buffer allows accurate imaging of actin filaments

Phalloidin (a fungal toxin of *Amanita phalloides* that binds specifically to filamentous actin), conjugated to a fluorophore, showed strong signal intensity with all four tested fixatives in infected (Figs. 1, 2) and control cells.

**Fig. 3:**
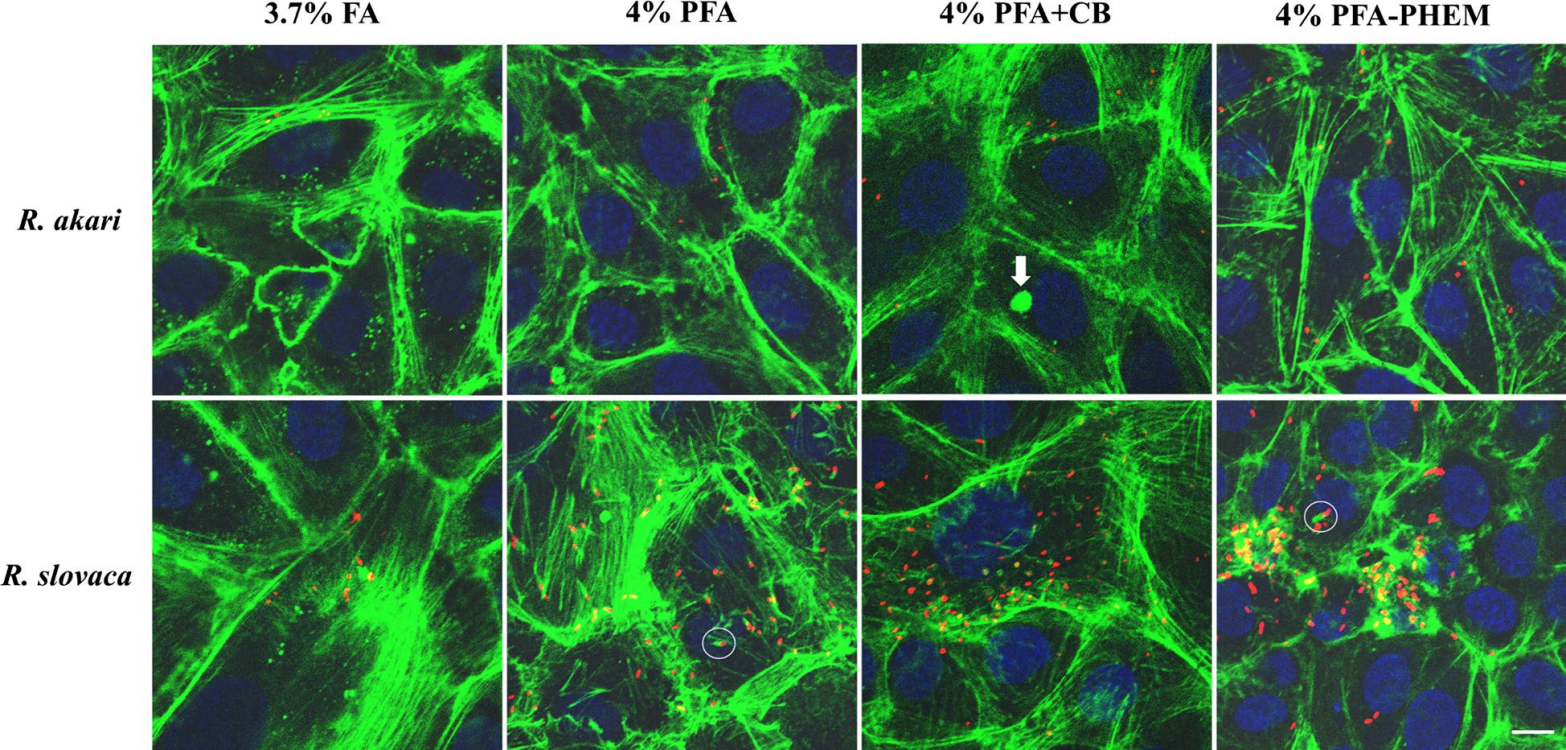
Merged images of infected Vero cells with rickettsiae and comparison of four fixative, summary of results. We ran parallel experiments on Vero cells infected with two species of rickettsiae, namely *R. akari* and *R*. *slovaca*, for optimization of fixatives to properly preserve actin cytoskeleton. We tested four fixatives, 3.7% formaldehyde (FA), 4% paraformaldehyde (PFA), 4% paraformaldehyde in the cytoskeletal buffer (PFA + CB) and 4% paraformaldehyde in PHEM buffer (PFA-PHEM). Formaldehyde conserved actin as discontinuous filaments compared to PFA-based fixatives. Unspecific artifact observed in 4% PFA + CB treatment is marked with an arrow. Our results suggest that 4% PFA-PHEM buffer gives the most accurate image of the actin cytoskeleton and simultaneously allows monitoring rickettsiae using actin-based motility during infection inside the host cells. Scale bar represents 10 µm

Formaldehyde fixation retained cytoskeletal structure the most poorly compared to other tested fixatives; filamentous actin appeared as beads on long fibers, giving it a broken appearance. Paraformaldehyde allowed revealing actin more faithfully as continuous filaments showing the overall shape of host cells. We were able to visualize “actin-tail” formation of *R. akari* and *R. slovaca* in both, 4% PFA- and PFA-PHEM-fixed infected host cells (highlighted in white circles in Figs. 1, 2, and 3, respectively).

Vero cell line, mock or infected, treated with 4% PFA + CB gave nonspecific signals more often than samples fixed with 4% PFA or PFA-PHEM (highlighted with a white arrow in Fig. 3) thus, we do not recommend this procedure.

Paraformaldehyde dissolved in PHEM buffer preserved the proper architecture of actin filaments forming a distinctive net throughout the cell. Therefore, we recommend using 4% PFA-PHEM buffer as the optimized procedure for immunofluorescent localization of *Rickettsia* sp. and “actin-tail” formation by these pathogens. However, we would like to note, that 4% PFA can also be used as a viable simplified alternative.

The summary of our findings in merged images of infected Vero cells with rickettsiae fixed with different cytoskeletal preserving solutions is shown in Fig. 3.

### Discussion

Here we present the effects of four aldehyde-based fixatives on Vero cells infected with rickettsiae (namely *R. akari* and *R. slovaca*) in vitro and provide a detailed optimized protocol for immune-based protein localization in three-color images (Figs. 1, 2 and 3).

The ability of rickettsiae to use actin-based motility was confirmed in several species of the SFG [3, 4, 12, 27]. *Rickettsia slovaca,* is commonly found in *Dermacentor* ticks in Slovakia [23, 26]. Comparing to *R. rickettsii* and *R. conorii*, which both typically exhibit a “comet tail” greater than 10 µm, *R. slovaca* seems to form it shorter, about 5 µm [10, 28]. Similarly, *R. akari* belonging to the transitional group of rickettsiae, promotes directional actin polymerization [12, 14].

Indirect immunofluorescence is widely used for the detection of pathogens in cell cultures [6, 15]. The first step in any successful immunocytochemical localization is the choice of fixative and fixation conditions [17].

Formaldehyde (the 1st alternative of fixation) works by chemically bonding adjacent macromolecules (neighbouring proteins) together, locking them in place with little morphological damage, so-called cross-linking [30].

Paraformaldehyde (the 2nd) is polymeric formaldehyde dissolved in water or a buffer. The free methanediols in the resulting solution are reactive with amine groups on proteins and other cellular structures that contain nitrogen. PFA crosslinks amino groups without changing the tertiary structure of proteins, therefore leaving most epitopes available for binding antibodies [7, 21]. Paraformaldehyde dissolved in the cytoskeletal buffer (PFA + CB) (the 3rd) with the inclusion of sucrose keeps the cell iso-osmotic, which also might help to preserve internal cell structures. Similarly, paraformaldehyde in PHEM buffer (the 4th) containing a high concentration of EGTA (10 mM or more) conserves structural integrity of all fibrous components of the cytoskeleton [22].

### Conclusions

We showed that an optimized fixation of actin filaments with 4% PFA-PHEM improved indirect immunofluorescence staining of rickettsiae. This allowed to accurately visualizing the actin-based motility of *R. akari* and *R. slovaca*. We suggest this approach as possibly applicable for imaging of other intracellular bacteria in host cells as well.

## Limitations

- choice of fixative
- choice of fixation conditions
- sensitivity of antibodies
- specificity for an antigen–antibody interaction.

## Data Availability

The datasets used and analysed during the current study are available from the corresponding author on reasonable request.

## Abbreviations

AG: ancestral group
FA: formaldehyde
IFA: immunofluorescence assay
MOI: multiplicity of infection
PBS: phosphate-buffered saline
PCR: polymerase chain reaction
PFA: paraformaldehyde
PFA + CB: paraformaldehyde in cytoskeletal buffer
PFA-PHEM: paraformaldehyde in PHEM buffer
PHEM buffer: containing PIPES, HEPES, EGTA and MgCl_2_
SFG: spotted fever group
TG: typhus group
TRG: transitional group
